# Impact of relationship status on psychological parameters in adults with congenital heart disease

**DOI:** 10.3389/fpsyt.2023.1260664

**Published:** 2023-11-17

**Authors:** Britta Stapel, Nicole Scharn, Tim Halling, Steffen Akkermann, Ivo Heitland, Mechthild Westhoff-Bleck, Kai G. Kahl

**Affiliations:** ^1^Department of Psychiatry, Social Psychiatry and Psychotherapy, Hannover Medical School, Hannover, Germany; ^2^Department of Cardiology and Angiology, Hannover Medical School, Hannover, Germany

**Keywords:** adult congenital heart disease, depression, anxiety, relationship status, cardiovascular disease

## Abstract

**Objective:**

Adult congenital heart disease (ACHD) is a growing disease entity, posing questions concerning psychosocial outcomes across the lifespan. Spousal relationships were shown to benefit cardiovascular and mental health in the general population. We assessed the association of relationship status with anxiety and depression in ACHD patients and determined whether patients considered disease-related concerns potential mediators of relationship problems.

**Methods:**

*N* = 390 ACHD patients were included. Self-report questionnaires were used to assess relationship status, ACHD-related relationship problems, socio-demographic variables, and depression and anxiety scores. Further, clinical parameters concerning the heart condition were determined.

**Results:**

*N* = 278 (71%) patients were currently in a relationship, while *N* = 112 (29%) were not in a relationship. Groups did not significantly differ regarding age, sex, and cardiovascular parameters. Two-way MANCOVA with relationship status and sex as independent variables, controlling for age, NYHA class, and NT-proBNP, showed an association of relationship status with depression, while sex was associated with anxiety. *N* = 97 (25%) patients reported disease-related adverse effects on a current or prior relationship. In detail, worries about body image (*N* = 57, 61%), own fears (*N* = 51, 54%), problems arising from wish to have children (*N* = 33, 35%), fears regarding a joint future (*N* = 29, 31%), partner’s fears or lack of understanding (*N* = 28, 30%), and sexual problems (*N* = 21, 22%) were cited.

**Conclusion:**

Relationships status was associated with depression, while sex was associated with anxiety in ACHD patients. Relationship status as well as potential relationship problems, and the importance of social support for mental and physical well-being, should be considered when treating ACHD patients.

## 1 Introduction

With a reported prevalence of 0.9–1% of live birth worldwide, congenital heart disease (CHD) represents the most commonly diagnosed congenital malformation in newborns (1, 2). With recent innovations in early diagnostic, interventional, and surgical procedures, the number of CHD patients that survive childhood and adolescence is steadily increasing. With up to 90% of patients reaching adulthood, factors that impact long-term cardiovascular disease (CVD) risk become increasingly important in patients with adult CHD (ACHD) (3, 4). In this regard, a current meta-analysis that assessed CVD risk of CHD survivors in later life found an increased risk for overall CVD, albeit the study was unable to pinpoint whether CHD constituted an independent risk factor or whether the association was confounded by a CVD risk factor profile among ACHD patients (5).

Next, to the increased CVD risk, ACHD patients display a significantly higher prevalence of psychiatric disorders when compared to the general population, with mood- and anxiety disorders being the most frequent (6–8). As psychiatric disorders are in turn associated with a heightened risk for CVD in the general population and symptomatic depression and anxiety are associated with adverse outcome measures, including rehospitalization and mortality, in patients with established CVD (9, 10), it is of clinical importance to identify modifiable factors that might negatively impact mental well-being in ACHD patients.

Various studies have evaluated the impact of (marital) relationship status on CV parameters and CVD risk in the general population. A twin study conducted in Sweden showed that living alone was associated with an increased CVD risk (11). Similar findings were reported in other countries and cultural regions (12). This association is further supported by a recent meta-analysis, which concluded that individuals that were not in a relationship had a higher CVD risk compared to married individuals (13). Next to the association with CVD risk, relationship status has also been shown to be associated with the outcome following a cardiac event, as individuals that lived alone were found to have an increased risk for all-cause mortality, CVD death, and myocardial infarction compared to individuals that lived in a marital relationship (14). While data regarding underlying mechanisms are limited, social support received from the partner is thought to reduce psychosocial stress and to thereby play an important role in mediating the beneficial effect of spousal relationships (15). Additionally, individuals in a relationship are thought to seek healthcare earlier and more often, to show better adherence to prescribed treatment, and to be more susceptible to healthier lifestyle behaviors (15).

Next, to CV parameters, relationship status has been associated with mental health measures. In this regard, spousal relationships have been described to be associated with protection from depression and anxiety in the general population (16, 17).

While research regarding relationship status in the context of ACHD is currently limited, a study assessing quality of life (QOL) in ACHD patients showed that next to older age, lack of employment, and higher New York Heart Association (NYHA) functional class, no marriage history was associated with lower QOL (18). Furthermore, feeling of loneliness was found as a common predictor of depression and anxiety in patients with ACHD (7).

As ACHD has been associated with an increased risk for CVD as well as for mood and anxiety disorders (5–8) and relationship status has been found to impact CV parameters and CVD risk as well as mental well-being in the general population (13), relationship status presents a relevant issue in the growing population of ACHD patients. Nevertheless, the association of relationship status with depression and anxiety has not been previously examined in ACHD patients and disease-related factors that might affect relationship quality and thereby stability of a spousal relationship have not been previously assessed.

Therefore, we examined relationship status and its impact on symptoms of anxiety and depression in a sample of ACHD patients. Additionally, we describe frequency and characteristics of disease-related relationship problems in these patients.

## 2 Materials and methods

### 2.1 Subjects

The presented data were generated as part of the ongoing PSYConHEART study that aims to establish morbidity and mortality factors in ACHD patients (19–22). Data collection took place from August 2020 to February 2021 at the outpatient clinic of the Department of Cardiology at Hannover Medical School. The study was conducted in accordance to the ethical guidelines of the 1975 Declaration of Helsinki and ethical approval was obtained from the local ethics committee at Hannover Medical School. All participants gave their written informed consent before entering the study. Inclusion criteria were a structural CHD, the ability to read and agree to the consent form and to read and answer the German versions of the relevant questionnaires, and an age ≥ 18 years. Exclusion criteria were pregnancy and instability of the cardiac condition. For our analyses that focused on the effect of spousal relationships, we considered respective literature that indicates that the main source for social support in adults are spousal relationships while in adolescents parental support was found to be the most important with regards to parameters of mental well-being (23). As data from the German Federal Statistical Office indicate a mean age of 23.6 years for young adults to leave their parental home in Germany in 2021 (24) we only included patients with an age ≥ 25 years in the analyses.

Data from *N* = 575 patients were obtained. After exclusion of cases with an age < 25 years, and cases that were missing data regarding relationship status, NYHA class, NT-proBNP, and/or hospital anxiety and depression scale (HADS) score. *N* = 390 cases were included in the study sample. Supplementary Figure 1 shows the sample selection process.

### 2.2 Cardiovascular evaluation

A senior cardiologist examined all patients included in the study during their routine check-up. The functional status of patients was determined by use of NYHA class. Cardiac morphology and function, including LVEF, were assessed by echocardiography. To classify the complexity of the underlying heart condition, the Bethesda scale was used to divide the congenital defect into “simple,” “moderate,” or “complex” (25). Additionally, number of thoracotomies was documented.

### 2.3 Assessment of psychosocial status

All participants answered a demographic survey that included relationship status (defined as an intimate spousal relationship that was marriage-like). Symptoms of depression and anxiety were assessed using the HADS, with the anxiety (HADS-A) and the depression (HADS-D) subscores being used (26). Additionally, patients were asked whether their heart defect had ever negatively impacted their relationship and to determine potential underlying issues, participants were asked to check either “yes” or “no” to the following suggested reasons: (1) negative body image, (2) own fears, (3) problems arising from wish to have children, (4) fears regarding a joint future, (5) fear or lack of understanding by the partner, and (6) sexual problems.

### 2.4 Statistical analyses

All statistical analyses were performed in SPSS 28 (IBM, Armonk, NY, United States). Shapiro Wilk Test was used for assessment of normality of data distribution. For group comparisons regarding anthropometric- and demographic data and CV parameters based on relationship status, non-parametric Mann–Whitney *U*-Test was used. Chi square test was performed for group comparisons of nominal data. To assess the association of relationship status and sex with depression and anxiety scores, two-way multivariate analysis of covariance (MANCOVA) was performed; HADS-D score and HADS-A score were imputed as dependent variables, relationship status and sex as independent variables, and age, NYHA class, and NT-proBNP as covariates. Sex was included as an independent variable based on dedicated literature that suggests distinct effects of relationship status on mental wellbeing in men and women in the general population (16). The respective covariates were included as prior studies reported conflicting results with regard to a potential association of disease severity and prognosis on psychological distress in ACHD patients (7, 27), and additionally an association of age, relationship status, and depression has previously been reported based on data from the general population (16). Two-tailed *p-*values are depicted and *p* ≤ 0.050 was considered statistically significant.

## 3 Results

### 3.1 Relationship status, and sociodemographic variables, and CV measures

An overview regarding sociodemographic factors and cardiac parameters of the study sample is provided in the Supplementary Results. Table 1 compares sociodemographic variables and CV measures in patients that reported to be currently in a relationship (*N* = 278 [71%]) to those that reported to be not in a relationship (*N* = 112 [29%]). Both groups did not significantly differ in any of the reported parameters.

**Table 1:** Comparison of sociodemographic variables and CV measures in ACHD patients based on current relationship status.

	Current relationship			
	No (*N* = 112)	Yes (*N* = 278)	Statistics	Value of *p*
Age (years)	38.8 ± 11.6	40.3 ± 11.0	*U* = 13892.5, *Z* = −1.664	*p* = 0.096
Female sex (*N* [%])	45 (40%)	141 (51%)	χ^2^(1) = 3.556, φ = 0.095	*p* = 0.059
Number of thoracotomies	1.6 ± 1.3	1.4 ± 1.1	*U* = 14645.0, *Z* = −0.793	*p* = 0.428
Bethesda scale			χ^2^(2) = 3.737, φ = 0.098	*p* = 0.154
Bethesda I (*N* [%])	9 (8%)	28 (10%)		
Bethesda II (*N* [%])	29 (26%)	96 (35%)		
Bethesda III (*N* [%])	73 (65%)	152 (55%)		
NYHA classification			χ^2^(3) = 0.833, φ = 0.046	*p* = 0.841
NYHA I (*N* [%])	75 (67%)	194 (70%)		
NYHA II (*N* [%])	27 (24%)	65 (23%)		
NYHA III (*N* [%])	9 (8%)	18 (7%)		
NYHA IV (*N* [%])	1 (0.9%)	1 (0.4%)		
LVEF (%)	54.8 ± 10.3	56.3 ± 8.6	*U* = 11625.0, *Z* = −1.133	*p* = 0.257
NT-proBNP (ng/L)	252.5 ± 322.3	295.4 ± 444.8	*U* = 14,965,0, *Z* = −0.599	*p* = 0.549
Psychotropic drug (*N* [%])	6 (5%)	15 (5%)	χ^2^(1) = 0.000, φ = 0.000	*p* = 0.997

If not indicated otherwise mean ± standard deviation (SD) is depicted and asymptotic two-tailed *p*-values are shown. *p* ≤ 0.05 was considered statistically significant. LVEF, left ventricular ejection fraction; NYHA class, New York Heart Association Functional classification, NT-proBNP, N-terminal pro b-type natriuretic peptide.

### 3.2 Association of relationship status and sex with depression and anxiety scores

**Figure 1:**
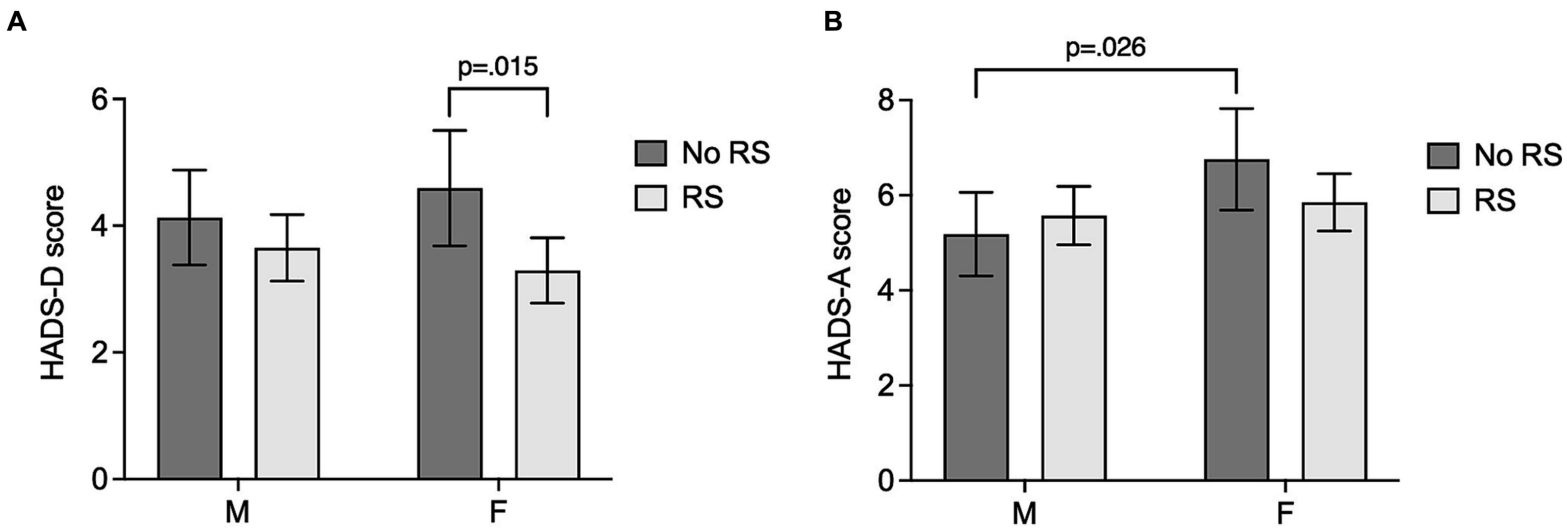
Depression and anxiety scores in ACHD patients in relation to relationship status and sex. Bar graphs depict estimated marginal means and 95% confidence intervals (calculated with age = 39.85, NYHA class = 1.39, and NT-proBNP = 283.12) of depressive symptoms measured by HADS-D score **(A)**, and anxiety symptoms indicated by HADS-A score **(B)**, dependent on relationship problems (No RS: no current relationship; RS: current relationship) and sex (M: male; F: female). Bonferroni-corrected two-tailed *p*-values for statistically significant pairwise group comparisons are depicted. *p* ≤ 0.050 was considered statistically significant.

Based on research indicating sex-specific effects of relationship status and social support on mental well-being (16), we assessed the association of relationship status and sex with HADS-D and HADS-A scores using two-way MANCOVA. To account for potential effects of age and disease severity, age, NYHA class, and NT-proBNP were included as covariates (7, 27). Two-way MANCOVA showed a statistically significant difference between relationship groups on the combined dependent variables [*F*(2, 382) = 4.352, *p* = 0.014, Wilk’s Λ = 0.978]. Additionally, sex had a statistically significant effect on the combined term [*F*(2, 382) = 4.371, *p* = 0.013, Wilk’s Λ = 0.978], while no significant interaction effect was found [*F*(2, 382) = 1.217, *p* = 0.297, Wilk’s Λ = 0.994]. *Post hoc* univariate ANCOVAs were conducted for both dependent variables. Results show a statistically significant difference between relationship groups for HADS-D scores [*F*(1, 383) = 6.330, *p* = 0.012, η^2^ = 0.016], while no significant difference for HADS-A score was found [*F*(1, 383) = 0.383, *p* = 0.537, η^2^ = 0.001]. Contrarily, a statistically significant difference between sexes was found for HADS-A scores [*F*(1, 383) = 5.020, *p* = 0.026, η^2^ = 0.013], while HADS-D scores did not significantly differ [*F*(1, 383) = 0.022, *p* = 0.882, η^2^ < 0.001]. Pairwise comparisons based on estimated marginal means using Bonferroni-corrected *post hoc* test showed a significant difference of HADS-D scores based on relationship status only in women (*p* = 0.015, *M*_Diff_ = 1.30, 95%-CI [0.255, 2.345]) but not in men (*p* = 0.308, *M*_Diff_ = 0.478, 95%-CI [−0.442, 1.398]). Additionally, increased anxiety scores in women compared to men were only observed in the no relationship group (*p* = 0.026, *M*_Diff_ = 1.575, 95%-CI [0.192, 2.957]), but not in the relationship group (*p* = 0.527, *M*_Diff_ = 0.279, 95% -CI [−0.586, 1.144]). Results are visualized in Figures 1A,B.

### 3.3 ACHD-related relationship problems

Given the observed protective effect of a spousal relationship on depressive symptomology in our sample, we assessed whether the underlying heart defect could have potential, adverse effects on a patient’s relationship. When asked, *N* = 97 (25%) of all ACHD patients in our sample reported that their heart disease had ever negatively impacted their relationship or prevented them from committing to a relationship, while *N* = 292 (75%) of patients reported no prior or current negative effect. Table 2 summarizes sociodemographic variables and CV measures of patients that had ever experienced a disease-associated adverse impact on their relationship compared to those who reported no previous or current impact. Patients that reported an adverse impact of their disease on their relationship did not significantly differ from those that were not affected with regard to current relationship status, age, or sex. However, patients that reported an adverse impact on their relationship presented with a higher disease severity indicated by a more complex underlying heart defect based on Bethesda class, a higher number of thoracotomies, a higher NYHA class and decreased LVEF. Additionally, patients that reported disease-related relationship problems had a prescription for at least one psychotropic drug more frequently.

**Table 2:** Comparison of sociodemographic variables and CV measures in ACHD patients based on reported disease-related relationship problems.

	Adverse impact on relationship			
	No (*N* = 292)	Yes (*N* = 97)	Statistics	*p*-value
Age (years)	40.4 ± 11.8	38.3 ± 9.2	*U* = 13157.0, *Z* = −1.048	*p* = 0.295
Female sex (*N* [%])	134 (46%)	51 (53%)	χ^2^(1) = 1.305, φ = 0.058	*p* = 0.253
Number of thoracotomies	1.4 ± 1.1	1.7 ± 1.3	*U* = 11611.5, *Z* = −2.554	*p* = 0.011
Bethesda scale			χ^2^(2) = 13.979, φ = 0.190	*p* < 0.001
Bethesda I (*N* [%])	28 (10%)	9 (9%)		
Bethesda II (*N* [%])	108 (37%)	17 (18%)		
Bethesda III (*N* [%])	153 (53%)	71 (73%)		
NYHA classification			χ^2^(3) = 13.744, φ = 0.188	*p* = 0.003
NYHA I (*N* [%])	215 (74%)	53 (55%)		
NYHA II (*N* [%])	61 (21%)	31 (32%)		
NYHA III (*N* [%])	15 (5%)	12 (12%)		
NYHA IV (*N* [%])	1 (0.3%)	1 (1%)		
LVEF (%)	56.3 ± 9.2	54.3 ± 8.8	*U* = 9301.5, *Z* = −2.360	*p* = 0.018
NT-proBNP (ng/L)	271.2 ± 416.0	320.1 ± 407.4	*U* = 12,810,5, *Z* = −1.409	*p* = 0.159
Psychotropic drug (*N* [%])	9 (3%)	12 (12%)	χ^2^(1) = 12.234, φ = 0.178	*p* < 0.001

If not indicated otherwise mean ± standard deviation (SD) is depicted and asymptotic two-tailed p-values are shown. *p* ≤ 0.05 was considered statistically significant. LVEF, left ventricular ejection fraction; NYHA class, New York Heart Association Functional classification, NT-proBNP, N-terminal pro b-type natriuretic peptide.

### 3.4 Reasons for ACHD-related relationship problems

To determine potential underlying reasons for disease-related relationship problems, patients were asked to answer “yes” or “no” to six suggested potential reasons as detailed in section 2.5. *N* = 94/97 patients that had report prior or current disease-related adverse effects on their relationship completed the respective questionnaire. Patients that reported problems in their relationship indicated underlying reasons with the following frequencies: negative body image (*N* = 57 [61%]), own fears (*N* = 51 [54%]), problems arising from wish to have children (*N* = 33 [35%]), fears regarding a joint future (*N* = 29 [31%]), fear or lack of understanding by the partner (*N* = 28 [30%]), and sexual problems [*N* = 21 (22%)]. *N* = 29 (31%) patients cited only one of these reasons to be applicable, however, most patients reported more than one reason for their relationship problems (*N* = 64 [68%]) (Supplementary Figure 2).

## 4 Discussion

One main result of our study is the finding that patients without a current relationship reported higher depression scores. Additionally, woman that were not in a current relationship also reported higher anxiety scores compared to men without a current relationship.

The second main finding of our study is that one fourth of patients in the present sample reported a negative impact of their CHD on a prior or current relationship. These patients were characterized by a more complex underlying heart condition and a more severe heart disease.

Our data indicate that patients with ACHD may benefit from a spousal relationship. In particular, our results suggest a greater benefit of being in a spousal relationship for women compared to men. In this regard, *post hoc* groupwise comparisons showed significant effects of relationship status on depression scores only in women but not in men and additionally, higher anxiety scores were detected in women that were not in a relationship compared to men with the same relationship status, while no effect of sex on anxiety scores was found in the relationship group.

While survival rates in patients have increased significantly over the last decades, ACHD patients with a moderate or complex underlying heart defect are often not cured and are confronted with medical complications and a shortened life expectancy (28–30). This might be associated with additional psychosocial challenges, which is reflected by the high frequency of depression and anxiety disorders in this patient population (6–8). In this regard, a study Kovacs and colleagues found that 50% of ACHD patients in the respective sample fulfilled criteria for at least one lifetime mood or anxiety disorder (7). Similarly, a prior study by our group found a prevalence of any mood disorder of 31% and of any anxiety disorder of 28% based on structured clinical interview in accordance to DSM-IV criteria (6). Therefore, the identification of factors that might protect from mood and anxiety symptoms is of importance.

Beneficial effects of spousal relationships on mental and on physical well-being have frequently been reported in the literature (31, 32). With regard to depression and depressive symptoms, various studies have found beneficial effects of marital relationships in the general population (16). An important factor that appears to confer beneficial effects of spousal relationships on protection from depression is perceived social support (23, 33). Perceived social support constitutes a subjective perspective of how individuals perceive the availability of material, psychological, and overall support offered by others (34). Perceived social support correlates well with various measures of mental health (35, 36). Of importance, social support is also characterized by the individuals that provide the support. In this regard, it is assumed that protective effects of social support vary depending on the provider, i.e., a spouse, relatives, or friends (23). A dedicated meta-analysis that reported on the association between social support and protection from depression found the strongest evidence for spousal support as a protective factor from depression in the adult population and especially emotional support was consistently found to be a protective factor (23).

Previous studies have found that ACHD patients experience mental health disorders, including depression and anxiety, with a higher prevalence than the general population (6–8). A study by Kovacs and colleagues found potential predictors for symptoms of depression and anxiety in these patients to be limited to feelings of loneliness and fear of negative evaluation as factors of social functioning, disease severity or functional class were not predictive (7). Contrarily, a recent publication reported a positive association of NYHA class and psychological distress (27). Our results are in line with the study by Kovacs et al. as no significant association of either NYHA class or NT-proBNP that were included as potential confounders in the MANCOVA, on HADS-D scores were detected in the present sample. While the impact of relationship status on depression and anxiety has not been previously evaluated in ACHD patients, prior studies have assessed relationship status in the context of quality of life in this patient population. Importantly, quality of life has been found to be significantly associated with anxiety and depression in ACHD patients (6). Previous studies have reported heterogenous effects regarding an association of marital relationship with QOL in patients with ACHD, with some studies reporting a significant association (18, 37, 38) while others failed to detect a significant effect (39). In line with our findings, a prior study reported that parameters of subjective functional status were only associated with the physical but not with the psychological domain of quality of life, while family support and psychological distress were common denominators for most quality of life domains including the psychological domain (40).

Of note, our data suggest greater effects of relationship status on depressive symptoms in women with ACHD compared to male patients. This is in contrast to data from the general population that suggest a greater benefit from marital relationships for men when compared to women. A study conducted with data from a series of cross-sectional national health surveys in Canada found modifying effects of age and sex on the relationship of marital status and depression (16). In this study, women that were single, widowed, or divorced were found to be less vulnerable to depression than men (16). The authors hypothesized that women more frequently utilize larger and stronger networks of social support while men often appear to rely on spousal support (16). However, this might not be the case in patients with CVD, as data from patients post acute myocardial infarction suggest that women experience lower levels of social support compared to men (41). Additionally, our results are in line with findings from Chen and colleagues that assessed determinants of quality of life in ACHD patients. The authors found sex-specific differences in the psychological domain of quality of life, which could be attributed to underlying psychosocial factors (40). Whether the observed sex-differences regarding the association of relationship status on protection from depressive symptoms are a specific feature of ACHD patients or whether other factors not investigated in our sample, including relationship satisfaction and the quality of social support by the spouse as well as other sources of social support, contribute to the observed effect will be subject of follow-up studies.

Overall, our findings expand data from previous studies that found that being in a (marital) relationship was associated with higher levels of psychological well-being, indicated by lower rates of depression and substance abuse in the general population as well as in patients that suffer from mental health problems, to ACHD patients (42).

In our sample, 71% of ACHD patients reported to be currently in a spousal relationship, which is comparable to a study from the Netherlands that reported 69% of patients to be in a spousal relationship (43). In that study, the rate of individuals in a relationship was significantly lower in the ACHD group than that observed in the respective control sample, in which relationship rate was 89% (43).

Given the association of relationship status and depressive symptomology in ACHD patients, and considering findings by others that commonly reported an effect of relationship quality, i.e., marriage dissatisfaction or conflict, on cardiovascular parameters, CVD risk, and mental well-being (15, 44, 45), it is of importance to identify potential disease-related problems that ACHD patients might experience with regard to their spousal relationship.

Our data show that one fourth of the ACHD patients in our sample reported that their disease had previously negatively impacted their relationship and those patients were characterized by a more complex underlying heart condition and a more severe heart disease.

When asked for reasons underlying their perceived relationship problems, most patients cited one of the suggested reasons. However, more than half of the patients cited more than one reason. Contrarily, to the finding that disease-related relationship problems were more frequent in patients with a more severe underlying heart disease, the frequency with which the different suggested reasons for these perceived problems were cited did not depend on disease severity. Additionally, the respective reasons were cited with similar frequencies by male and female patients, with the exception of “problems arising from wish to have children” that was reported significantly more often by women (data not shown).

Overall, our data suggest protective effects of being in a spousal relationship on depressive symptoms in patients with ACHD. In light of literature that reports that ACHD patients are in spousal relationships at a lower rate than the general population (43), it appears of importance to identify factors, including those associated with the underlying cardiac defect, that might adversely affect relationship quality and stability. Our data show that a considerable percentage of patients has previously found their heart disease to adversely impact their relationship or prevented them from entering a relationship. Most patients cited at least one disease-related reason that could be attributed to the patient. Therefore, it might be considered to address relationship status as well as potential relationship problems, and the importance of social support for mental and physical well-being, when treating patients with ACHD.

### 4.1 Limitations

Our study has several limitations that should be considered. We only present cross-sectional data, which does not allow for temporal or causal inference. We did not assess whether patients that were not in a relationship were single, separated, or widowed. Therefore, we did not investigate any potential differences in these subgroups with regards to depression and anxiety scores, which is of importance, as literature suggests distinct effects on depression scores (16). Additionally, data regarding quality of social support by the spouse, as well as other sources of social support were not assessed in our sample. Finally, we did not assess current relationship quality, which could have impacted depression scores as literature suggests adverse effects of relationship conflict or dissatisfaction on mental health parameters (46–48).

## Data availability statement

The raw data supporting the conclusions of this article will be made available by the authors, without undue reservation.

## Ethics statement

The studies involving humans were approved by the local Ethics Committee at Hannover Medical School. The studies were conducted in accordance with the local legislation and institutional requirements. The participants provided their written informed consent to participate in this study.

## Author contributions

BS: Formal analysis, Visualization, Writing – original draft. NS: Formal analysis, Writing – original draft. TH: Investigation, Writing – review & editing. SA: Investigation, Writing – review & editing. IH: Formal analysis, Writing – review & editing. MW-B: Conceptualization, Formal analysis, Project administration, Supervision, Writing – review & editing. KK: Conceptualization, Formal analysis, Project administration, Supervision, Writing – review & editing.
