# Supplementary material for: Impact of relationship status on psychological parameters in adults with congenital heart disease
Source: Front Psychiatry. 2023 Nov 17;14:1260664. doi: 10.3389/fpsyt.2023.1260664 (PMC10690776; doi:10.3389/fpsyt.2023.1260664)
Supplement: Supplementary file 1 [file Data_Sheet_1.PDF]

Supplementary Material

1 Supplementary Figures

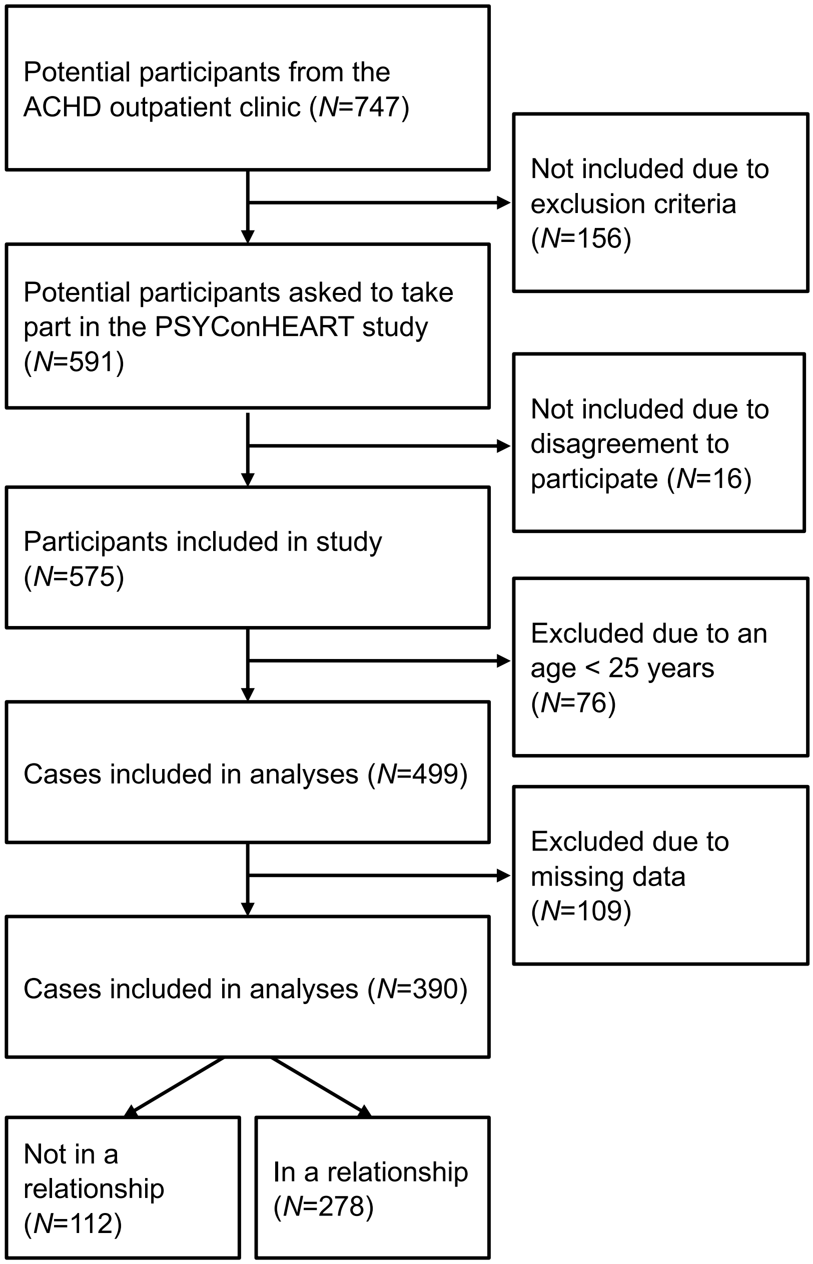

Supplementary Figure 1. Scheme detailing recruitment of patients and reasons for exclusion from the analyses.

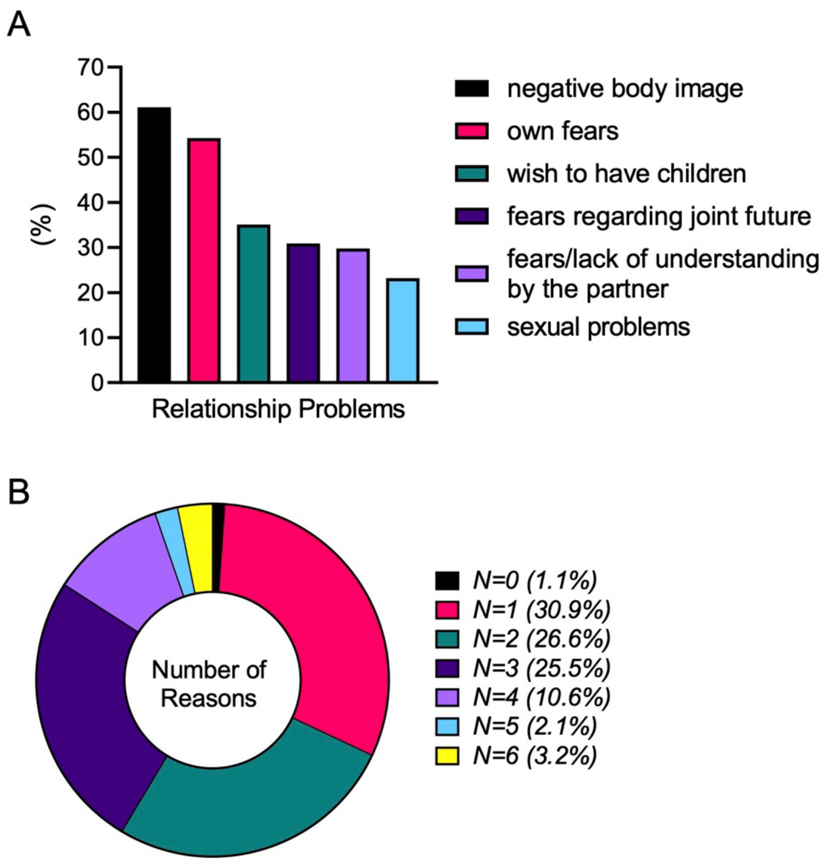

Supplementary Figure 2: Frequencies of reported reasons for perceived relationship problems in ACHD patients. (A) Frequencies of the reasons for relationship problems; multiple answers were possible. (B) Frequencies of number of reasons for relationship problems.

2 Supplementary Results

2.1 Demographic data and cardiac parameters of the study sample

Mean age of the sample was 40 ± 11 years and $N = 186$ (48%) of the included patients were female. Most patients in the sample presented with no or mild symptoms of chronic heart failure (NYHA class I: $N = 269$ [69%]; NYHA class II: N=92 [24%]), while patients that presented with moderate to severe symptoms were less frequent (NYHA class III: $N = 27$ [7%]; NYHA class IV: N=2 [0.5%]). Regarding the complexity of underlying congenital heart defect, patients most frequently presented with complex defects ($N = 225$ [58%]), followed by moderate defects ($N = 125$ [32%]) and simple defects ($N = 37$ [10%]) in accordance to the Bethesda classification.
